# Molecular Simulation Study on the Wettability of a Surface Texturized with Hierarchical Pillars

**DOI:** 10.3390/molecules28114513

**Published:** 2023-06-02

**Authors:** Kiduk Kim, Seyong Choi, Zhengqing Zhang, Joonkyung Jang

**Affiliations:** 1 Department of Nanoenergy Engineering, Pusan National University, Busan 46241, Republic of Korea; 2 State Key Laboratory of Separation Membranes and Membrane Processes, School of Chemical Engineering and Technology, Tiangong University, Tianjin 300387, China

**Keywords:** molecular dynamics simulation, Cassie-Baxter, Wenzel, hydrophobic surface, hierarchical pillars

## Abstract

By using molecular dynamics simulation, we investigate the wettability of a surface texturized with a periodic array of hierarchical pillars. By varying the height and spacing of the minor pillars on top of major pillars, we investigate the wetting transition from the Cassie–Baxter (CB) to Wenzel (WZ) states. We uncover the molecular structures and free energies of the transition and meta-stable states existing between the CB and WZ states. The relatively tall and dense minor pillars greatly enhance the hydrophobicity of a pillared surface, in that, the CB-to-WZ transition requires an increased activation energy and the contact angle of a water droplet on such a surface is significantly larger.

## 1. Introduction

*Superhydrophobic* surfaces find wide applications including anti-icing [1], anti-frosting [2], water collection [3], oil-water separation [4], drag-reducing [5,6], self-cleaning [7], prevention of corrosion [8,9], solar cells [10,11], and light-emitting diodes [12,13], just to name a few. Over the years, the wettability of a pillared surface has been extensively studied both experimentally [3] and theoretically [14,15,16,17]. Researchers have explored surfaces texturized with pillars that are diverse in shape and size. Surfaces texturized with cylindrical, rectangular, or dome-shaped pillars have been most commonly studied [18,19,20,21,22].

The current consensus view is that a superhydrophobic surface should provide a static *contact angle* (CA) > 150° for a water droplet deposited on it. Additionally, a sliding angle < 10° and a hysteresis of (advancing and receding) CA < 10° are required for the high mobility of a water droplet on a surface (dynamic hydrophobicity).

Besides CA, another critical information pertaining to the wettability (hydrophobicity) of a pillared surface is whether the gaps between pillars are wetted by a water droplet deposited on top. The wetted and de-wetted states of the *inter-pillar gap* are, respectively, called the *Wenzel* (WZ) [23] and *Cassie–Baxter* (CB) [24] states. Attempts have been made to find out the optimal geometry (shape, size, and spacing of pillars) of a pillared surface, which preferentially provides the CB, instead of WZ state.

**Figure 1:**
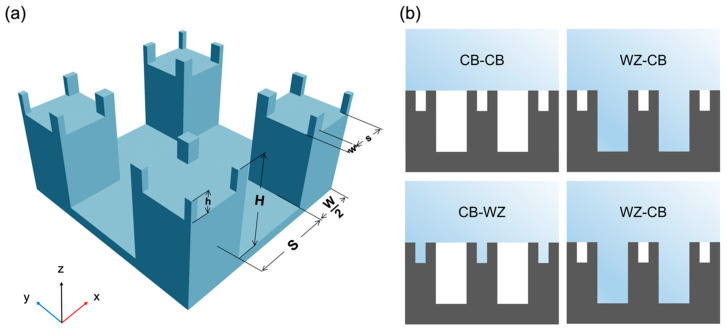
(**a**) Periodic simulation cell of the present surface texturized with hierarchical pillars. The height, width, and interpillar spacing of major pillars are represented by H, W, and S, respectively. On top of each major pillar, we constructed multiple minor pillars of h, w, and s in height, width and interpillar spacing, respectively. (**b**) Schematic diagram for four distinct wetting states possible for a surface texturized hierarchical pillars: CB-CB, WZ-WZ, CB-WZ, and WZ-CB states.

It is well known that a natural superhydrophobic surface, such as that of lotus leaf or rose petal, is texturized with an array of pillars with a *hierarchical* structure; each major pillar is covered by multiple minor pillars smaller in size (Figure 1a). In the case of a surfaced texturized with dual-scale pillars, there can be four distinct wetting states, depending on whether the gaps between the minor and major pillars are wetted or not, giving rise to the CB-CB, CB-WZ, WZ-CB, and WZ-WZ states (Figure 1b).

Experimental and computational efforts have been made to study the superior hydrophobicity of a surface patterned with hierarchical pillars. Yang et al. reported the CA of a water droplet on a flat surface increased from 71.6° to 104° and 145° by fabricating single-scale and dual-scale pillars on the flat surface, respectively [25]. Nikosokhan et al. obtained a CA of 160° by patterning cobalt-based hierarchical pillars on a surface [26]. By using molecular dynamics (MD) simulation, Kwon et al. found that the presence of hierarchical pillars greatly increases the CA from that of a surface texturized with nonhierarchical pillars. Additionally, the mobility of a droplet also enhanced with the hierarchical pillars [27].

In spite of the extensive prior studies on the wettability of a surface texturized with hierarchical pillars, we still poorly understand the *metastable* state and *transition* state (TS) existing between the CB and WZ states. The structures and relative stabilities of these intermediate states are quite important to understand the wettability of a pillared surface. The (de)wetting transition can be kinetically trapped in the meta-stable state due to the free energy barrier(s) needed to surmount the TS (s). The molecular details and free energies involved in this activated process are largely unknown.

Previously, we have studied, by using the MD simulation, the CB-to-WZ transition for a surface engraved with dual-scale trenches [28]. However, such a trenched surface differs from a pillared surface that is grooved along two lateral directions crossing each other. Others reported MD simulations for surfaces engraved with hierarchical pillars [14,15,17] but the focus was on the CA of water droplet deposited on such a surface.

Herein, we report on the MD simulation on a surface texturized with hierarchical pillars, which emulates a natural superhydrophobic surface. We uncover the intermediate metastable states and TSs of the CB-to-WZ transition for a surface patterned with hierarchical pillars. By identifying these intermediate states, we estimate the free energy barriers and feasibilities (kinetics) for the wetting and de-wetting transitions. We show that hierarchical pillars exhibit a superior hydrophobicity by making the CB state more stable than the WZ state, and by lowering the free energy barrier for the de-wetting transition. Our results provide fundamental insights into the design of a novel hydrophobic surface utilizing hierarchical pillars.

## 2. Results and Discussion

We simulated a surface texturized with a periodic array of hierarchical pillars as follows. Out of the face-centered cubic lattice of carbon [29], we engraved four major pillars with a height H and a width W, separated from each other by spacing S (Figure 1a). We simulated major pillars with various heights, Hs (=7.1, 8.9, 10.7, 12.5, 14.3, and 19.6 Å) by fixing S to 23.2 Å and W to 48.2 Å. On top of each major pillar, we constructed multiple minor pillars with a height h and a width w, each of which is separated by an interpillar spacing, s (Figure 1a). An infinite array of hierarchical pillars was emulated by applying the periodic boundary conditions (PBCs) to the simulation cell (Figure 1a).

In the case of a pillar patterned with hierarchical pillars, we fixed H to 14.3 Å and w to 5.35 Å. We examined the effects of varying the *relative height* of the minor pillars h* defined as h* = h/H. We varied h* as 0.5 and 0.75 by fixing the *relative spacing* of minor pillars s*, defined as s* = s/S, to 0.23. We also studied both the dense and sparse minor pillars by simulating s* = 0.23 and 0.69, respectively (h* = 0.25 for both cases). The sparse and dense minor pillars provided 9 or 25 minor pillars per major pillar, respectively. For comparison, we also simulated surfaces texturized with nonhierarchical pillars.

**Figure 2:**
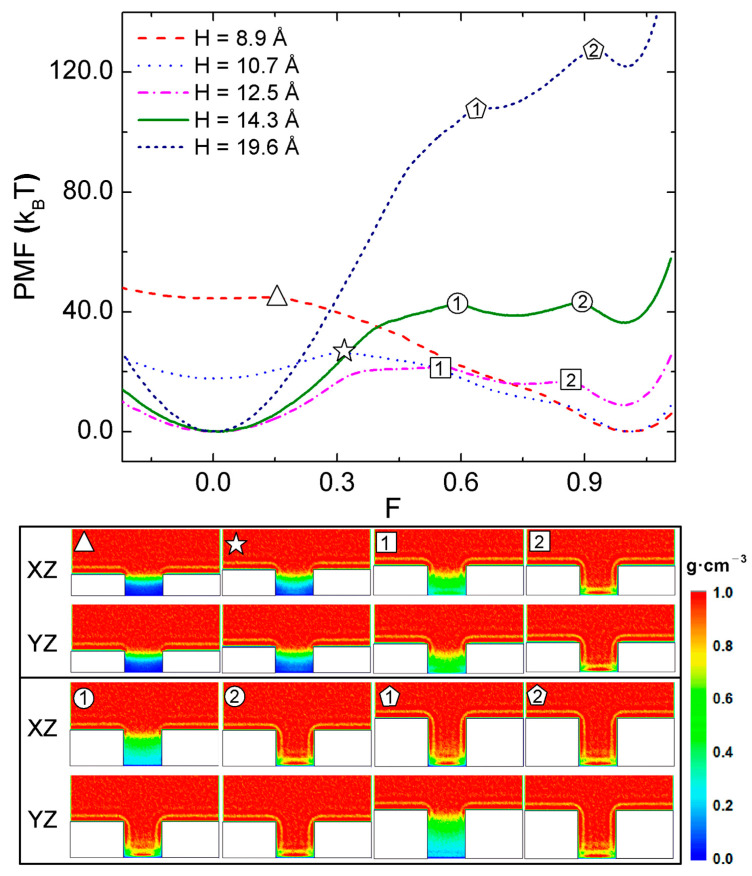
Free energy profiles of the CB-to-WZ wetting transitions of the surfaces texturized with nonhierarchical pillars. At the top, the PMF is plotted vs. the filling level F for surfaces with pillars with heights Hs of 8.9, 10.7, 12.5, 14.3, and 19.6 Å. The transition states, defined as the maxima in the PMF curves, are marked as symbols. The density profiles of water at the transition states are shown at the bottom, marked as different symbols in the PMF curves. In each panel, two cross sections of the density profile taken along the XZ and YZ planes are drawn as color maps.

We first examine the (CB-to-WZ) wetting transition for a surface texturized with nonhierarchical pillars. By changing H as 8.9, 10.7, 12.5, 14.3, and 19.6 Å with S fixed to 23.2 Å, we investigated how the PMF and water density depend on the filling level F (Figure 2). As shown in the top of Figure 2, two minima of PMF near F = 0 and F = 1 are noticeable, corresponding to the CB and WZ states, respectively. The relative stabilities of CB and WZ states depend on the height of pillars; for the surfaces with relatively short pillars with H = 8.9 and 10.7 Å, the WZ state is more stable than CB state (near F = 0) because the global minimum of PMF curve is found near F = 1. Therefore, the CB state is a metastable state and the interpillar gap is more likely to be filled with water for these short pillars. On the other hand, for the surfaces with relatively tall pillars, H = 12.5, 14.3, and 19.6 Å, the CB state is more stable than the WZ state, signifying that the interpillar gap tends to be de-wetted thermodynamically. In these cases, the CB (WZ) state is stable (metastable).

We marked the maxima in the PMF curves (Figure 2, top) as different symbols. These maxima correspond to the TSs in the CB-to-WZ transition. The heights of these maxima (measured from the minima on the left) can be regarded as the *activation energies* of the wetting transitions. As H varies as H = 12.5, 14.3, and 19.6 Å, the activation energy increases 21.641, 42.969, and 127.763 $k_{B}T$, respectively; as the aspect ratio of pillar increases, the wetting transition to the WZ state becomes increasingly difficult. One can conclude that increasing the aspect ratio of a pillar enhances the stability of the CB state, and therefore the super-hydrophobicity of the present pillared surface. It can be also seen that two TSs exist in the cases of surfaces with relatively tall pillars (H = 12.5, 14.3, and 19.6 Å).

At the bottom of Figure 2, we illustrate the structures of various TSs marked in the PMF curves (Figure 2, top). In each panel, we show two cross sections of the density profile of water taken along the XZ and YZ planes (note the surface normal is along the Z axis). In the cases of relatively tall pillars with H = 14.3, and 19.6 Å, water penetrates into the interpillar gap non-uniformly as the density profiles projected onto the XZ and YZ planes are different; for example, the first TS for H = 14.3 Å has different density profiles taken along the XZ and YZ planes. Along the XZ plane, the liquid water barely penetrates into the interpillar gap, while the water touches the bottom of the interpillar gap along the YZ plane. Similarly, an anisotropic penetration of water is seen in the first TS for H = 19.6 Å. Therefore, two TSs existing between the CB and WZ states originate from the anisotropic penetration of water into the interpillar gap. The first TS corresponds to the penetration along one direction, and the second TS to the penetration along the other direction. The present anisotropic penetration of water was also found the previous study on the wetting transition [30]. Interestingly, in the case of H = 12.5 Å, the TS looks identical along the XZ and YZ planes. Nevertheless, two TSs are found for the CB-to-WZ transition.

**Figure 3:**
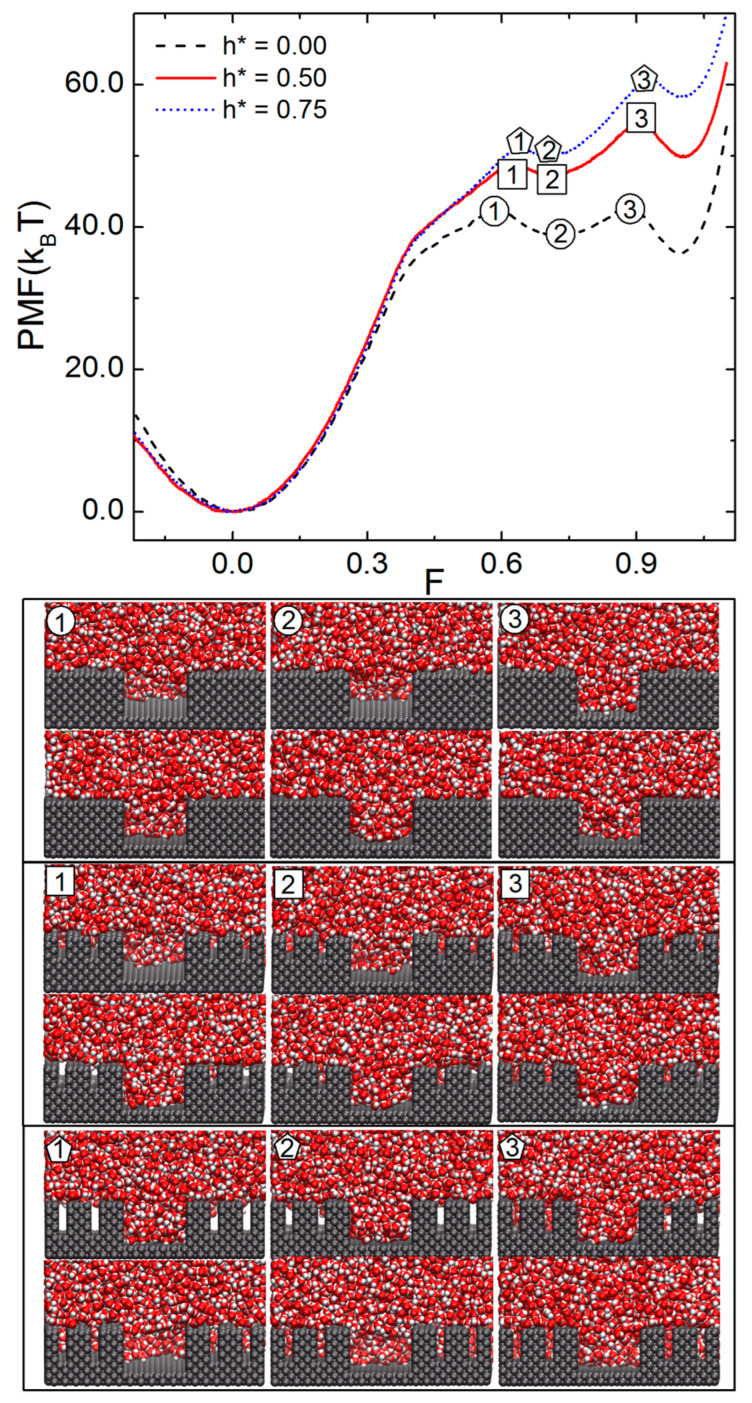
Free energy profiles for the wetting transitions of surfaces with the hierarchical pillars. The PMF is plotted vs. the filling level F for minor pillars with relative heights, h*s (h* = h/H), of 0.0, 0.5 and 0.75. The representative snapshots of water at the transition states, marked as different symbols in the PMF curves, are shown at the bottom. In all the cases shown here, s* is fixed to 0.231.

We have seen, for nonhierarchical pillars, that increasing the height of pillars makes the CB state more stable than the WZ state, thereby enhancing the activation energy for the CB-to-WZ transition. In this perspective, we investigate the effects of varying h* for surfaces with hierarchical pillars. To do so, we fixed H = 14.3 Å and s* = 0.231 and simulated the cases for h* = 0, 0.5, and 0.75. Figure 3 plots the PMF vs. F and the simulation snapshots of various TSs marked in the PMF curves. For all three PMF curves shown, two TSs exist between the CB and WZ states. This again originates from the fact that water penetrates into the interpillar gap anisotropically. For example, in the first TS of the PMF curve for h* = 0.5, water penetrates halfway down to the bottom of the gap between major pillars along one direction, but nearly touches the bottom in the other direction. One can also see that the degree of penetration into the minor-interpillar gap also differs depending on the direction.

The present anisotropic penetration of water is a non-ergodic event on the timescale of the present MD simulation (ns). Statistically, the penetration along the X and Y directions should be identical. Dynamically or kinetically, however, the symmetry of X and Y directions is broken temporarily due to the thermal fluctuation of water. Such a non-ergodicity of molecular dynamics has been often observed in the MD simulation; for example, the velocity distribution of molecules evaporating from a nanodroplet of water did not follow the Maxwell–Boltzmann equation [31]. The diffusivity of a liquid water was found to depend on the initial condition of the MD simulation [32]. It is left as a future work to investigate whether the present nonergodicity persists on a much longer timescale (micro or milliseconds).

The presence of minor pillars greatly affects the wetting properties of the present pillared surface. More specifically, by increasing h* from 0 to 0.5 and 0.75, the WZ state becomes increasingly more unstable than the CB state. The activation energy for the CB-to-WZ transition increases by increasing h*. The free energy barrier of the first TS (measured from the CB state) varies at 42.87 $k_{B}T,$ 49.41 $k_{B}T$, and 51.15 $k_{B}T$, as h* changes from 0, 0.5, and 0.75, respectively. The second activation energy for the transition from the intermediate metastable state to the WZ state (transition from label 2 to label 3 in the PMF curve) varies between 4.27 $k_{B}T$, 7.65 $k_{B}T$, and 11.00 $k_{B}T$, respectively, by changing h* as 0, 0.5, and 0.75. Therefore, as h* increases, the CB-to-WZ transition becomes increasingly difficult.

**Figure 4:**
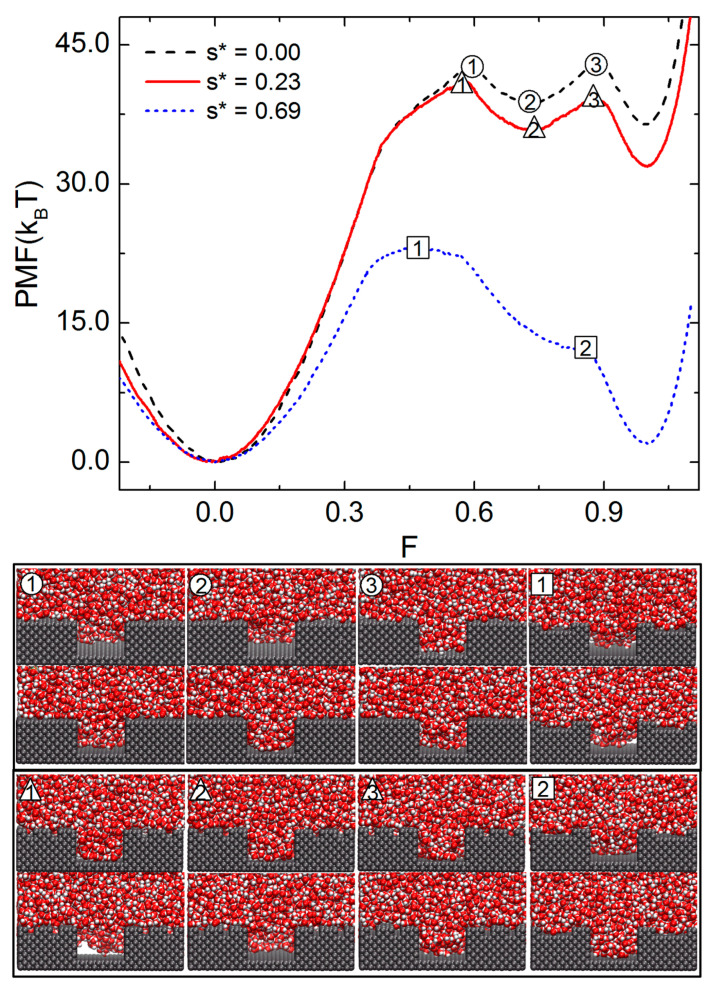
Free energy profiles for the wetting transitions of surfaces patterned with hierarchical pillars. The PMF is plotted vs. the filling level F for minor pillars s* = 0.0, 0.23 and 0.69. The representative snapshots of water at the transition states, marked as different symbols (numbers in triangles or squares) in the PMF curves, are shown in the bottom. In all three cases, h* is fixed to 0.25.

We now demonstrate how the presence of minor pillars can deteriorate the hydrophobicity of the present pillared surface. Figure 4 illustrates the PMF profiles and various TSs and metastable states simulated by setting s* to 0, 0.23, and 0.69. Here, H is fixed 14.3 Å and h* = 0.25. By increasing s*, the first TS appears at a lower value of F; the first TS is found at F = 0.59, 0.57, and 0.45, respectively, for the cases of s* = 0.0, 0.23, and 0.69. In the case of s* = 0.23, the PMF vs. F curve qualitatively resembles that of the surface with nonhierarchical pillars, s* = 0. Quantitatively, however, the free energy barrier of the first TS decreases from 42.8 $k_{B}T$ to 41.43 $k_{B}T$ by increasing s* from 0 to 0.23. The barrier of the second transition (label 2 to 3) also decreases from 4.27 to 3.50 $k_{B}T$. With further increasing s* to 0.69, the free energy of the WZ state is significantly lowered, only slightly above that of the CB state. Additionally, the free energy barrier for the first TS noticeably lowers to 23.15 $k_{B}T$. Additionally, the free energy barrier for the second TS is negligible. The snapshots of the TSs in this case show that water penetrates into the gap between major pillars nearly isotropically. In summary, the presence of hierarchical pillars with sparse minor pillars reduces the hydrophobicity of the present pillared surface. The previous studies also reported that a secondary structure of a pillar can decrease the hydrophobicity of a pillared surface.

**Figure 5:**
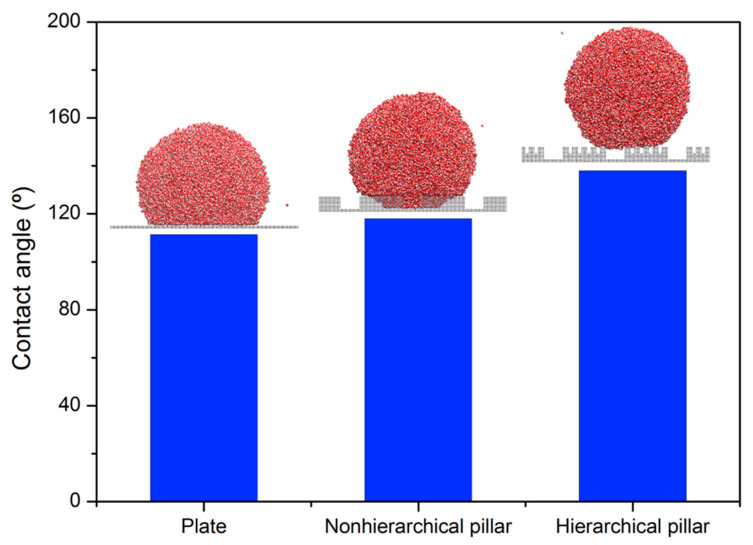
Variation in the CA of a water droplet with changing the texture of a surface. The CAs are shown for the flat surface and the surfaces patterned with nonhierarchical and hierarchical pillars. We set H = 14.3 Å and S = 23.2Å for the pillared surfaces. In the case of the surface with hierarchical pillars, h* = 0.5 and s* = 0.23.

Above, we have seen that the presence of hierarchical pillars with dense minor pillars increases the hydrophobicity of a surface by destabilizing the WZ state and by increasing the activation energies for the CB-to-WZ transition. To confirm this conclusion, we further check the CAs of water droplets deposited on the flat surface and if the surfaces are texturized with nonhierarchical and hierarchical pillars. In order to eliminate the effects of line tension on the CA, we simulate a large droplet made of 8000 water molecules [33]. As shown in Figure 5, a water droplet composed of 47,940 water molecules (14 nm radius) is deposited on three surfaces described above. By fixing H = 14.3 Å, we simulated a surface with non-hierarchical pillars and a surface with hierarchical pillars of h* = 0.5 and s* = 0.23. The CAs are 111, 118, and 138 degrees, respectively, for the flat surface, the surface with nonhierarchical pillars, and the surface with hierarchical pillars. Therefore, the existence of hierarchical pillars provides the highest CA. Note the water droplet on the hierarchical pillars takes the CB state, while the water droplet penetrates down into the interpillar gap of the nonhierarchical pillars (shown in the middle of Figure 5). This increase in CA by 20° through introducing minor pillars corroborates our conclusion that the existence of hierarchical pillars with dense minor pillars enhances the hydrophobicity of a surface.

## 3. Simulation Methods

A macroscopic water droplet resting on top of a pillared surface was simulated as follows. A surface texturized with pillars were carved out from the face-centered cubic lattice of carbon [29] with a lattice space of 3.567 Å. Each pillared surface was covered by a layer of water with a thickness of 40.0 Å (6200 water molecules). In order to prevent the fictitious interaction between water and bottom of the surface, due to the PBCs applied in simulation, we added a 3.0–5.0 nm vacuum slab on top of the water layer. We used the extended-simple-point-charge model (SPC/E) [34] to describe the intermolecular interaction of water. The interaction between water oxygen atoms were modeled by using the Lennard–Jones (LJ) potential, $U^{LJ}\left(r_{ij}\right)=4\varepsilon_{ij}\left[{\left(\frac{\sigma_{ij}}{r_{ij}}\right)}^{12}-{\left(\frac{\sigma_{ij}}{r_{ij}}\right)}^{6}\right]$, where $\varepsilon_{ij}$ and $\sigma_{ij}$ are the well-depth parameters and the size parameters set to 0.6502 kJ mol^−1^ and 3.166 Å, respectively. The interactions between carbon and water oxygen atoms were also modeled by using the LJ potential with $\varepsilon_{ij}$ is 0.4389 kJ mol^−1^ and $\sigma_{ij}$ is 3.190 Å. The long-range Coulomb interactions between point charges were treated with the particle–particle-particle–mesh (PPPM) method [35]. We cut off both the LJ and Coulomb interactions at a distance of 12.0 Å.

The MD trajectories were propagated using the velocity Verlet algorithm [36] with a time step of 2.0 fs. We simulated rigid water molecules by using the SHAKE algorithm [37] and all the carbon atoms were frozen. A 3.0 ns long MD simulation was run in the canonical ensemble (NVT) at 300 K by using the Nosé–Hoover thermostat [38,39]. The final configuration obtained in the NVT MD simulation was used as the initial condition for the subsequent calculation of the *potential-of-mean force* (PMF) below. MD methods were implemented by using LAMMPS (22Aug2018) software [40].

We calculated the PMF by varying the collective variable D, defined as the height of the center-of-mass of water measured from the bottom of the surface. D is an input parameter controlled in our calculation of PMF, in order to continuously tune the degree of wetting (from that of the CB state to that of the WZ state). In simulating the PMF vs. F, D is restrained to a specific value. By varying D in a series of such restrained MD simulations, F changes from 0 to 1. Using the umbrella sampling [41], we set up D to a target value ranging from 29.0 to 23.8 Å, from 30.4 to 24.4 Å, from 33.2 to 25.4 Å, from 34.4 to 26.4 Å, from 37.6 to 26.8 Å, from 44.0 to 28.2 Å, (with a decrement of 0.2 Å) for H = 7.1 Å, 8.9 Å, 10.7 Å, 12.5 Å,14.3 Å, and 19.6 Å, respectively. For each D in the PMF curve, we ran a 6.0 ns-long NVT simulation at 300K by imposing the harmonic-bias potential with 200.0 kcal/(mol·Å^2^), which constrains D to a target value. The initial 0.2 ns was discarded for equilibration. The PMF profile was extracted using the weighted histogram analysis method [40,42]. Finally, D was converted to the *filling fraction* F defined as:

$$F=\frac{D_{CB}-D}{D_{CB}-D_{WZ}}$$

where D_CB_ and D_WZ_ were Ds corresponding to the CB and WZ states, respectively. By construction, F = 0 and 1 for the CB and WZ states, respectively. The CB and WZ states were defined as those located at the local or global minima of the PMF curve. The PMF calculation method described above was implemented by using the LAMMPS combined with the PLUMED package [42].

We further calculated the CA of a water droplet for a selective set of the present pillared surfaces. The water droplet on a pillared surface (Figure 5) was simulated by using a normal MD simulation without restraining D. To do so, a spherical droplet of 47,940 H_2_O molecules (14.0 nm in diameter) was equilibrated by running an NVT simulation at 300 K for 2.0 ns. The equilibrated droplet was then positioned 1.0 nm above a pillared or flat surface. A 16.0 ns-long NVT simulation was run for the water droplet. The initial 1.0 ns was discarded for equilibration. The CA was calculated by employing the method of Khalkhali et al. [43]: in this method, a convex hull algorithm was used to approximate the liquid–vapor interface of a droplet as a collection of small interconnected triangles. The CA was further obtained from the surface normal vectors of these triangles. This method obviates construction of the density profile of water by means of the three-dimensional binning of molecules, which can be computationally intensive. Additionally, the shape of a liquid droplet does not have to be assumed in this method. The computational efficiency of this method is much better than that of a conventional method, especially for a big droplet. The present large droplet, made of 47,940 water molecules, prevents the line tension effect, which provides an increased CA with decreasing the size of a droplet [33,44].

A pillared surface might contain defects and atomic-scale roughness that should be considered to be modelled more realistically. However, the present ideal geometry without any defects should be a reasonable starting point and should capture the essential features of the wetting transition of a pillared surface. Unlike a typical hydrophilic surface terminated by surface hydroxyls, we modelled a nonpolar carbon surface which should have minimal effects of water dissociation or the electrostatic interaction of water with the surface. Similarly, Walther et al. [45] reported that the electrostatic interaction has a negligible effect on the structure of a liquid water surrounding a carbon nanotube. It is left as a future work to implement the electrostatic water–surface interaction by using sophisticated force fields, such as those reported by Heinz et al. [46] and by Pramanik et al. [47].

## 4. Conclusions

By using MD simulation, we studied the CB-to-WZ wetting transition for a surface texturized with hierarchical pillars. Various TSs and metastable states existing between the CB and WZ states were found. Owing to the anisotropic penetration of water into the interpillar gap, two distinct TSs existed in the CB-to-WZ transition. The presence of the minor pillars on top of major pillars could increase or decrease the hydrophobicity of a pillared surface. Relatively tall and dense minor pillars greatly enhanced the hydrophobicity of a pillared surface; the CB state became more stable than the WZ state and the activation energy for the CB-to-WZ transition increased. This enhanced hydrophobicity was also confirmed by the increased CA of a water droplet on such a surface. On the other hand, relatively short and sparse minor pillars actually decreased the hydrophobicity of a pillared surface from that of a surface with nonhierarchical pillars. The present findings should be helpful for furthering our understanding of how the presence of hierarchical pillars affects the wettability of a surface.

## Data Availability

Data sharing not applicable.
